# Efficacy and Safety of the Combination of Diclofenac and Thiocolchicoside in the Treatment of Low Back Pain and Other Conditions: Systematic Review of the Literature

**DOI:** 10.3390/healthcare13060677

**Published:** 2025-03-20

**Authors:** Ioannis Oikonomou, Karolina Akinosoglou

**Affiliations:** 1 Department of Medicine, University of Patras, 26504 Rio, Greece; akin@upatras.gr; 2 Department of Internal Medicine and Infectious Diseases, University General Hospital of Patras, 26504 Rion, Greece

**Keywords:** diclofenac, thiocolchicoside, low back pain, musculoskeletal pain, systematic review, pain management, combination therapy

## Abstract

**Background/Objectives**: Low back pain (LBP) is a leading cause of disability worldwide. Diclofenac, a non-steroidal anti-inflammatory drug (NSAID), and thiocolchicoside, a muscle relaxant, are commonly combined to target inflammation and muscle spasm. However, the efficacy and safety of their combination remain under discussion. This systematic review evaluates the efficacy and safety of diclofenac-thiocolchicoside therapy for LBP and other musculoskeletal conditions. **Methods**: A systematic review was conducted following PRISMA guidelines. Eligible studies included randomized controlled trials (RCTs) and observational studies comparing diclofenac-thiocolchicoside combination with placebo, monotherapy, or alternative treatments. A search was performed in PubMed, Scopus, and relevant websites, identifying articles published up to 30 September 2024. Studies from trial registries were excluded. Risk of bias was assessed using Revised Cochrane Risk of Bias for randomized trials (RoB 2) for RCTs and the Newcastle-Ottawa Scale (NOS) for observational studies. Evidence certainty was evaluated with the Grading of Recommendations, Assessment, Development, and Evaluations (GRADE) framework. Results were visualized using Robvis, tables, and graphs. **Results:** Of 393 identified records, 9 studies (1097 patients) met the inclusion criteria. Seven RCTs reported significant pain reduction and functional improvement with combination therapy compared to placebo or active controls. However, study heterogeneity, dosage variations, and risk of bias limited comparability. Adverse events (AEs) included gastrointestinal (GI) discomfort and drowsiness, though no severe complications were consistently reported. **Conclusions**: Despite methodological limitations, the diclofenac-thiocolchicoside combination demonstrates promising efficacy for acute LBP and musculoskeletal pain management. However, there is no clear evidence of its clinical superiority over other available treatments, due to study heterogeneity and potential biases. Rigorous, standardized research with larger sample sizes and consistent methodologies is essential to definitively establish the efficacy and safety of diclofenac-thiocolchicoside, providing clearer guidance for clinical decision-making.

## 1. Introduction

Pain is one of the most frequently reported health issues encountered both in clinical environments and within the general population. Managing it effectively remains a significant, unresolved global challenge in the field of medicine. Acute pain typically stems from a specific injury or illness, whereas chronic pain is increasingly regarded as a standalone medical condition [1].

Musculoskeletal pain encompasses a group of conditions characterized by inflammation and degeneration within the musculoskeletal system [2]. The most common types include LBP, neck pain, and pain related to osteoarthritis and rheumatoid arthritis [3]. Musculoskeletal pain is widespread and represents a significant challenge for individuals and healthcare systems alike, cutting across genders, age groups, and sociocultural boundaries. It is recognized as one of the leading causes of pain and physical disability, affecting hundreds of millions globally [4].

LBP, specifically, is a highly prevalent condition and a leading cause of disability worldwide. It affects many individuals at some point in their lives, with significant variation in prevalence depending on demographic and methodological factors [5]. One systematic review estimated the global point prevalence of activity-limiting LBP at 11.9% and a one-month prevalence at 23.2% [6]. Chronic LBP (CLBP), defined as pain persisting for 12 weeks or more, affects 5–10% of individuals with acute LBP, often leading to substantial personal and societal burdens [5].

The prevalence of LBP varies by age and sex. It is most commonly reported in individuals aged 40–80 years and is more prevalent in women than men [6]. Additionally, prevalence tends to increase with age, peaking in the sixth decade of life. In Brazil, for instance, the prevalence of CLBP among older adults reaches 25.4% [5]. Such age-related trends underscore the challenges posed by an aging global population, as the number of individuals experiencing LBP is expected to rise dramatically in the coming decades [6]. LBP imposes a significant economic burden due to healthcare costs, lost productivity, and disability compensation [7,8,9,10].

Despite its prevalence and impact, the management of LBP remains complex due to its multifactorial nature. Most cases are classified as nonspecific, lacking a definitive pathoanatomical diagnosis, which complicates treatment [11].

LBP is influenced by a range of individual, physical, and psychological factors, many of which contribute to its onset and chronicity [12,13]. Lifestyle factors such as obesity, smoking, and poor general health also play significant roles [14]. Psychosocial elements, including depression [12], anxiety [12], job dissatisfaction [13], and low social support, further exacerbate the risk, as psychological distress often correlates with heightened pain perception and persistence [7,12,13,14,15,16,17,18].

LBP management aims to reduce pain, improve function, and prevent chronicity through a combination of nonpharmacologic and pharmacologic strategies. For acute LBP, self-care measures such as maintaining physical activity, applying heat, and avoiding prolonged bed rest are emphasized [19,20,21,22]. Nonpharmacologic therapies, including spinal manipulation, acupuncture, and massage, provide modest benefits for some patients, depending on accessibility and patient preference [11]. Pharmacologically, NSAIDs and skeletal muscle relaxants are preferred for their effectiveness in reducing pain and disability, though their benefits are typically small and short-lived [11].

CLBP requires a more multidisciplinary approach, often integrating physical therapy [23], exercise [24], and cognitive-behavioral therapy [25,26] to address physical and psychosocial contributors to pain. Adjunct pharmacologic treatments include NSAIDs, antidepressants, and occasionally opioids. However, opioids are reserved for cases of severe, refractory pain due to their potential for dependence and other adverse effects [27]. A systematic review of pharmacologic treatments revealed only small benefits from NSAIDs, muscle relaxants, and opioids in chronic LBP, with variable evidence on safety and tolerability [11]. Noninvasive interventions, such as graded exercise and patient education, remain core components, although evidence supporting their long-term efficacy varies [28].

Diclofenac, a derivative of benzene acetic acid, is an NSAID known for its analgesic, antipyretic, and anti-inflammatory properties. It functions as a non-selective, reversible, and competitive inhibitor of cyclooxygenase (COX), thereby hindering the conversion of arachidonic acid into prostaglandin precursors. This process reduces the production of prostaglandins involved in pain, inflammation, and fever [29]. Thiocolchicoside is a semisynthetic compound derived from colchicoside, a natural glucoside found in the plant *Gloriosa superba*. It has been utilized in clinical settings for over 35 years as a muscle relaxant, as well as an anti-inflammatory and analgesic agent. Due to these effects, thiocolchicoside has been widely employed in managing various orthopedic, traumatic, and rheumatologic conditions [30]. Its mechanism of action involves functioning as a competitive antagonist of GABAA receptors while also inhibiting glycine receptors with comparable potency and exerting a lesser effect on nicotinic acetylcholine receptors [31]. Biochemical studies indicate that thiocolchicoside promotes central nervous system (CNS) depression, leading to muscle relaxation [30].

The combination of Diclofenac sodium and Thiocolchicoside is well-established and extensively documented. Decades of clinical use have shown no pharmacokinetic interactions between these active ingredients. Furthermore, a phase I bioequivalence study has demonstrated that an intramuscular fixed-combination product containing Diclofenac sodium and Thiocolchicoside is bioequivalent to the combined administration of Diclofenac 75 mg (Voltaren) and Thiocolchicoside 4 mg (MuscoRil) mixed in a single syringe [32]. This compatibility is further supported by the Summary of Product Characteristics (SmPC) of MuscoRil as authorized in Italy [33].

Various medicinal products containing Diclofenac and Thiocolchicoside as separate active pharmaceutical ingredients (APIs) have been approved and marketed in both European Union (EU) and non-EU countries. Diclofenac-containing products are primarily indicated for treating a broad spectrum of musculoskeletal conditions, including LBP, while Thiocolchicoside is indicated as an adjuvant therapy to alleviate muscle spasms associated with musculoskeletal disorders, such as acute or chronic rheumatic diseases.

Currently, a single EU-authorized product combines Diclofenac and Thiocolchicoside in a solution for intramuscular injection at a strength of 75 mg + 4 mg per 4 mL. This product, marketed under the brand name Dicloside, was authorized in Greece in 2022.

This systematic review aims to evaluate the efficacy and safety of diclofenac and thiocolchicoside combination therapy for LBP and other musculoskeletal conditions, addressing gaps in existing research. Specifically, we aim to assess the efficacy of the combination by measuring pain reduction, functional improvement, and patient-reported outcomes, examine the safety of the combination by analyzing AE incidence and severity associated with this combination therapy, and synthesize existing evidence by identifying study heterogeneity and limitations to guide clinical recommendations and future research.

## 2. Materials and Methods

### 2.1. Eligibility Criteria

This systematic review included both interventional (e.g., randomized controlled trials [RCTs]) and observational studies (e.g., cohort studies, case-control studies). Interventional studies were selected to evaluate the efficacy and safety of the combination therapy under controlled conditions, while observational studies provided real-world evidence on its use.

Studies were eligible if they included adult participants (≥18 years) diagnosed with LBP or other musculoskeletal conditions for which the combined use of diclofenac and thiocolchicoside was indicated. There were no restrictions on participants’ sex, ethnicity, or comorbidities.

The review focused on studies investigating the combined use of diclofenac (as sodium, potassium, or other salts) and thiocolchicoside. Studies were included if the combination was administered via any route (e.g., oral, intramuscular) and at any dosage.

Eligible studies compared the combination therapy with monotherapy using either diclofenac or thiocolchicoside, other treatments for LBP or musculoskeletal pain, placebo, or no treatment.

The primary outcomes were measures of efficacy, including pain relief (e.g., Visual Analogue Scale (VAS) scores), functional improvement, and patient-reported quality of life. Secondary outcomes included the incidence and severity of AEs associated with the combination therapy.

Studies published in scientific journals were eligible, provided they presented original research data (Table 1). Abstracts, case reports, conference proceedings, unpublished data, and clinical trial registries were excluded.

### 2.2. Information Sources and Search Strategy

A comprehensive search was conducted in October 2024 to identify studies evaluating the combined use of diclofenac and thiocolchicoside for managing LBP and other musculoskeletal conditions. The search included articles published up to 30 September 2024. The databases searched were PubMed and Scopus. The websites Academia and ResearchGate were also searched. The keywords “diclofenac” and “thiocolchicoside” were used, combined with the Boolean operator “AND” to ensure the retrieval of studies focusing on both agents.

To ensure the inclusion of high-quality evidence, only studies with full-text availability were considered, while abstracts alone were excluded. No language restrictions were applied, allowing for a broad and inclusive search. The reference lists of identified articles were not manually reviewed, and grey literature was not included in this review.

### 2.3. Data Selection and Collection Process

The data selection and collection process for this systematic review followed a dual-reviewer approach with independent screening. One reviewer conducted the primary search in the specified databases, while the second independently cross-checked search results. Titles and abstracts of the identified articles were screened for relevance, and full texts were assessed for final inclusion based on pre-specified eligibility criteria. Disagreements between reviewers were resolved through discussion. No automation tools were used in the selection process.

For data extraction, both reviewers independently reviewed the full text of each included study and manually extracted key details, including study design, population characteristics, interventions, analysis methodologies, and reported outcomes. Extracted data were compared between reviewers, and any discrepancies were resolved through discussion. Data were compiled into structured summary tables, manually created by one reviewer and independently verified by the other.

All identified articles were downloaded in PDF format for detailed examination. Given the relatively limited number of included studies, a manual data collection approach was deemed sufficient. The databases were searched multiple times to minimize the risk of missing relevant studies.

No automation tools were used at any stage of data selection or extraction.

### 2.4. Data Items

The data extraction process focused on predefined primary and secondary outcomes, as well as key study characteristics relevant to the systematic review.

The primary outcomes included measures of pain reduction and functional improvement. Pain intensity was assessed using validated tools such as the VAS and the Numerical Rating Scale (NRS), while functional improvement was evaluated through standardized instruments like the Oswestry Disability Index (ODI) and the Finger-to-Floor Distance (FFD) test. Whenever multiple pain or functional assessments were reported within a study, preference was given to the most commonly used validated metric to ensure consistency in the analysis. Data were collected for all reported time points, including baseline, post-treatment, and follow-up assessments.

The secondary outcomes primarily addressed safety and patient-reported measures. AEs were documented and categorized into gastrointestinal side effects, such as nausea, vomiting, and gastritis, and neurological effects, including dizziness, drowsiness, and sedation. Other reported AEs, including allergic reactions or unexpected serious complications, were also recorded. Additionally, data on the use of rescue medication, global treatment satisfaction, and treatment discontinuation rates were extracted when available.

In addition to outcome measures, several study-level variables were collected to facilitate a comprehensive synthesis of the evidence. These included details on the study design, distinguishing between RCTs and observational studies, as well as sample size, patient demographics, and diagnostic criteria. Information on the intervention was carefully extracted, including drug formulation (oral, intramuscular, or other), dosage, and treatment duration. Data on comparator groups were also retrieved, particularly whether the diclofenac and thiocolchicoside combination was compared against placebo, diclofenac monotherapy, thiocolchicoside monotherapy, or alternative treatments.

To address unclear study classifications, an attempt was made to contact the corresponding author of one study to clarify its true study design [34]. Although the paper was labeled as an observational study, the methodology closely resembled that of a clinical trial, making its classification ambiguous. Further concerns were raised due to the journal in which the study was published, as it appears on Beall’s list of predatory journals, raising additional questions about its credibility. However, no response was received from the author, and the study’s classification remained based on the information available in the published manuscript.

For other missing or unclear information, a structured approach was applied. In cases where essential study details such as sample size or intervention specifications were missing, the study was excluded from synthesis. When studies presented only summary statistics without individual time-point data, only the reported summary measures were extracted.

### 2.5. Risk of Bias and Certainty of Evidence

The risk of bias and quality of the included studies were assessed using validated tools tailored to the study design. For RCTs, the RoB 2 tool [35] was utilized, evaluating five domains: (1) randomization, (2) deviations from intended interventions, (3) missing outcome data, (4) outcome measurement, and (5) selection of reported results (Supplementary Materials). Observational studies were evaluated using the NOS [36] focusing on selection, comparability, and outcome or exposure assessment. These tools ensured a systematic and structured assessment of bias across the included studies.

Each study was assessed independently by both reviewers, following the guidance provided in the Cochrane Handbook for Systematic Reviews of Interventions [37]. Disagreements were handled through discussions.

For the RCTs, overall risk-of-bias judgments were generated as “low”, “some concerns”, or “high” risk, based on the RoB 2 methodology. Similarly, the NOS ratings provided a clear indication of study quality in terms of methodological rigor and risk of bias. A summary of the RCT assessments was visualized using the RoBvis tool to enhance clarity and accessibility. This figure highlights the distribution of risk across domains and provides an at-a-glance overview of the methodological quality of the trials.

Publication bias could not be assessed using funnel plots, nor was it formally tested through Egger’s test, since the number of studies included in the review was less than ten.

In addition to visual representation, a narrative description of the bias assessments is provided in the text, detailing the key domains and specific concerns identified within each study. This dual presentation format ensures a comprehensive understanding of the bias and quality of the included studies and facilitates a critical interpretation of the findings. A synthesis of main efficacy outcomes was conducted.

Finally, the GRADE approach was used to determine the certainty of evidence for the main extracted outcomes.

### 2.6. Effect Measures

The primary outcomes assessed in this systematic review were measures of efficacy, including pain relief, functional improvement, and patient-reported quality of life. Pain intensity was primarily evaluated using validated tools such as the VAS, which provides a quantitative measure of pain reduction over time. Functional improvement was commonly assessed through measures like the ODI, FFD, and other standardized mobility tests. These metrics allowed for a comprehensive evaluation of the impact of the combination therapy on both pain severity and physical function.

Secondary outcomes included the incidence and severity of AEs associated with the combination therapy. These were categorized into GI, neurological, and other adverse effects, reflecting the known safety profiles of diclofenac and thiocolchicoside. Patient satisfaction with treatment and the requirement for rescue medication were also reported in some studies, providing additional insights into the overall acceptability and effectiveness of the therapy. Together, these outcomes facilitated an in-depth exploration of the therapeutic value and safety of the diclofenac and thiocolchicoside combination.

The primary outcome measure for pain intensity was evaluated using VAS scores (0–1 cm or 0–100 mm). Studies presented mean baseline VAS scores and final VAS scores, allowing for the calculation of the mean difference by subtracting the final score from the baseline score. Some studies directly reported mean differences, while others provided raw VAS scores.

### 2.7. Data Synthesis

Given the significant heterogeneity among the included studies in terms of design, population characteristics, interventions, and outcome measures, a narrative synthesis approach was employed. Quantitative meta-analysis was not feasible due to the small number of studies included, the variability in study methodologies, the inconsistency in reported data, and differences in comparator groups and assessment tools.

The narrative synthesis followed a structured approach to summarize and interpret the findings. Studies were grouped based on their primary focus, such as randomized controlled trials versus observational studies, and their reported outcomes. Common themes and trends were identified, including the consistency of pain relief and functional improvement compared to baseline values observed across studies, as well as the safety profiles of the therapies. Variations in findings were examined in the context of study quality, risk of bias, and differences in interventions or populations.

The synthesis aimed to integrate the available evidence while acknowledging the limitations posed by study heterogeneity, guiding meaningful interpretation and highlighting areas for future research.

## 3. Results

### 3.1. Study Selection

**Figure 1:**
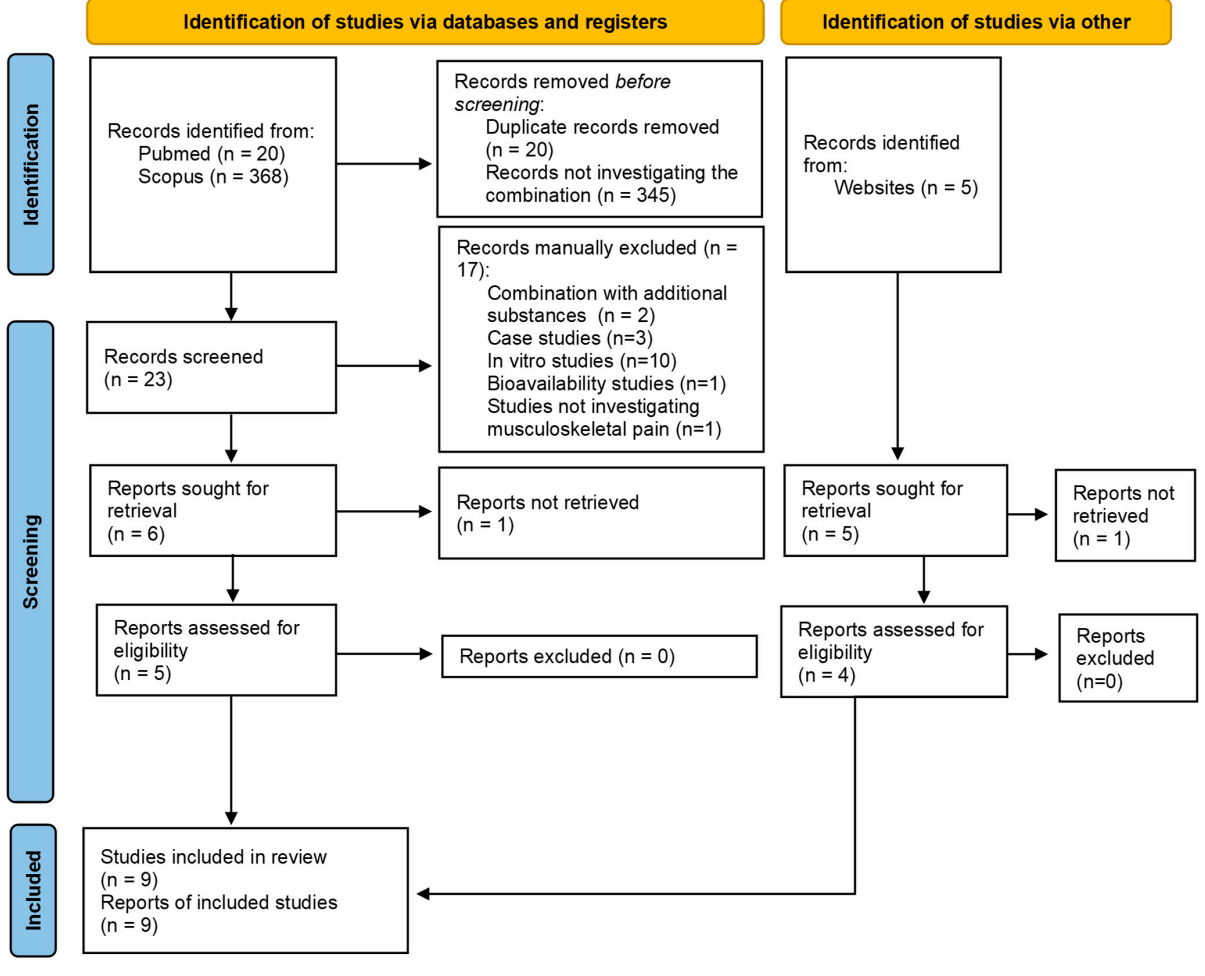
PRISMA 2020 flow diagram of study selection [48].

Out of 393 total reports identified, 9 met the inclusion criteria (Figure 1); 7 were RCTs [38,39,40,41,42,43,44], 1 was a retrospective cohort [45], and 1 was a prospective cohort [34]. Initially, the study by Akhter and Saddiq [38] was considered an observational study, as stated within the paper. However, the methodological description lacked sufficient clarity. Research results identified a systematic review and meta-analysis that confirmed that this was, in fact, an RCT, while also defining the intramuscular injection as the dosage form and route of administration of the interventions [46]. Additionally, the methodological description provided by Asawari et al. [34] also exhibited a similar challenge, as it was not possible to definitively determine whether the study was truly observational or aligned more closely with an RCT. Attempts to contact the study author for clarification were unsuccessful. Consequently, for the purposes of this review, the study was classified as observational, as stated within the paper.

One study initially met the inclusion criteria [47], however, only its abstract was available. The full text was requested via ResearchGate but was never provided. Therefore, this study was not included in the review.

### 3.2. Study Characteristics

The RCTs reviewed focus on the management of acute LBP and involve diverse methodologies, patient populations, and geographical contexts. For instance, Altan et al. conducted a randomized, double-blind study in Turkey to evaluate the efficacy of phonophoresis with Diclofenac and Thiocolchicoside gel compared to standard ultrasound therapy [39]. Similarly, Ambrish et al. compared Thiocolchicoside-Diclofenac to Eperisone-Diclofenac in a randomized trial in India [41]. Studies by Iliopoulos et al. and Sproviero et al. assessed intramuscular combinations of Diclofenac and Thiocolchicoside versus monotherapy in Greece and Italy, respectively [42,44]. The study by Landázuri et al. evaluated an oral fixed-dose combination in Ecuador [43]. These trials involve varying sample sizes, ranging from 60 to over 200 participants, focusing on patients with acute moderate to severe LBP.

The observational studies included in this systematic review also span diverse geographical and clinical contexts. Asawari et al. investigated the effectiveness of treatments in orthopedic inpatients at Bharati Vidyapeeth University, Pune, India, over a six-month period [34]. Meanwhile, Meloncelli et al. carried out their study in a single center in Italy, targeting patients with acute LBP [45]. Although conducted in different regions, all studies shared the goal of assessing the efficacy of various pharmacological interventions for LBP.

### 3.3. Risk of Bias in Studies

**Figure 2:**
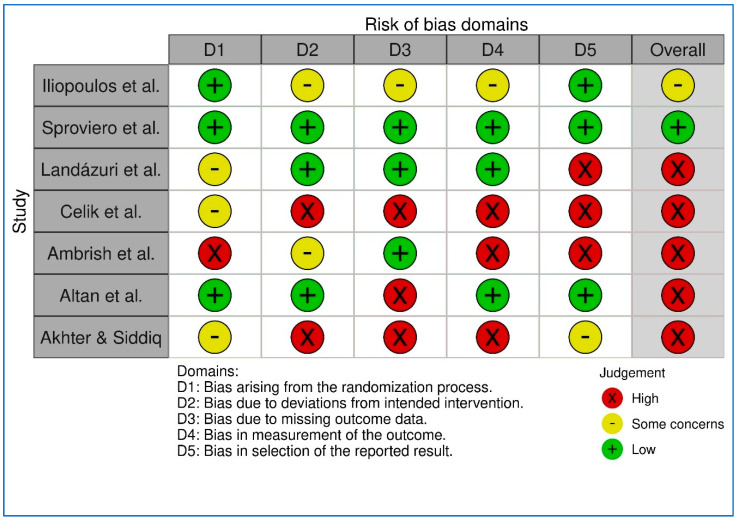
RobVis Traffic Light Table [38,39,40,41,42,43,44].

**Figure 3:**
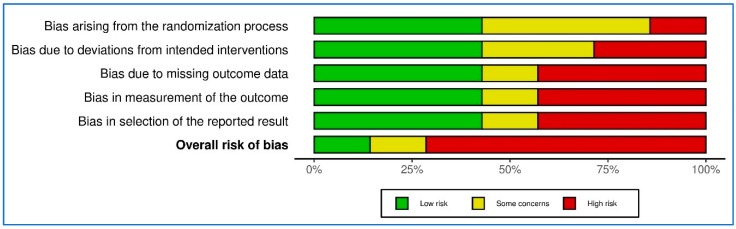
RoBvis summary graph.

Visual summaries were generated using RoBvis tools (Figure 2 and Figure 3) for the assessment of RCTs. A table summary was generated for the assessment of the observational studies (Table 2).

### 3.4. Efficacy Outcomes

The efficacy of the combination of Diclofenac and Thiocolchicoside was assessed in all included studies (Table 3). The primary outcomes included pain intensity reduction, functional improvement, and muscle spasm relief, with studies employing validated tools such as VAS, ODI, FFD, and Schober test. Notably, VAS was the most consistently utilized tool across the majority of studies.

The study by Altan et al. [39] assessed the efficacy of the combination of diclofenac and thiocolchicoside delivered via phonophoresis (PP) in comparison to conventional ultrasound (US) therapy with a non-pharmaceutical gel in patients with acute LBP. Sixty participants were randomly allocated into two groups: Group 1 (PP using diclofenac and thiocolchicoside gel) and Group 2 (conventional US with placebo gel). Efficacy outcomes were evaluated at baseline, Week 2 (W2), and Week 6 (W6) using key metrics including the VNS, ODI, and Schober test. At rest, both groups exhibited significant reductions in VNS scores at W2 and W6 compared to baseline. At W2, the median VNS score decreased from 5 to 2 in Group 1 and from 3 to 2 in Group 2. By W6, Group 1 showed a further reduction to a median of 1 (range: 0–6), while Group 2 remained at 1 (range: 0–4). The intergroup comparison at W6 demonstrated significantly greater improvement in Group 1 (*p* = 0.041). During movement, Group 1 achieved a reduction in VNS scores from a baseline median of 8 to 3.5 at W2 and 3.0 at W6. In Group 2, scores decreased from 8 to 5 at W2 and 4 at W6. The reduction was significantly greater in Group 1 at W6 (*p* = 0.023).

At W2, both groups showed significant functional improvement in the ODI, with Group 1 achieving a median ODI reduction of 11 points (range: −29 to −1) and Group 2 a reduction of 15 points (range: −26 to 2). By W6, Group 1 exhibited a more pronounced improvement, with a median reduction of 22 points (range: −31 to −3), compared to a reduction of 13 points (range: −29 to −2) in Group 2 (*p* = 0.007).

Both groups demonstrated significant within-group improvements in Schober test scores at W2 and W6 relative to baseline. Group 1 showed a median increase of 0.5 cm (range: 0–2) by W6, while Group 2 showed a similar increase. However, the intergroup comparison did not reveal statistically significant differences at either time point.

The study by Ambrish et al. [41] provided a comparative analysis of the efficacy of diclofenac combined with either thiocolchicoside (Group A) or eperisone (Group B) in patients with LBP. The investigation included 60 participants randomly allocated into two groups, with outcomes assessed over a 7-day period. The primary efficacy metrics included the FFD, Lasegue’s Sign, the VAS for pain, and a Global Assessment Scale of therapeutic response. Statistical significance was defined as *p* < 0.05.

On Day 1, the mean FFD was 24.3 ± 11.41 cm in Group A and 16.6 ± 13.454 cm in Group B. By Day 7, these values significantly improved to 6.83 ± 3.63 cm and 2.67 ± 2.155 cm, respectively.

The intergroup comparison on Day 7 revealed a statistically significant difference favoring Group B (*p* = 0.000). The improvement percentages were 71.9% in Group A and 83.9% in Group B, indicating greater efficacy of the eperisone-diclofenac combination in reducing muscle spasm and improving flexibility.

At baseline (Day 1), 46.7% of Group A patients exhibited moderate hypertonia, which improved to normal in 46.7% of cases by Day 7. Conversely, Group B began with 46.7% of patients showing mild hypertonia, with 80% achieving normal tone by Day 7. The improvement in Lasegue’s Sign was statistically significant (*p* = 0.016), with Group B showing superior normalization of lumbar tone compared to Group A.

The mean VAS score on Day 1 was 6.37 ± 1.63 in Group A and 6.43 ± 1.79 in Group B. By Day 7, scores decreased to 2.17 ± 1.31 in Group A and 1.33 ± 1.63 in Group B. Both intragroup improvements were statistically significant (*p* = 0.000), but the intergroup difference on Day 7 also favored Group B (*p* = 0.033).

At the conclusion of the study (Day 7), 60% of Group A patients were rated as having a “good” response to treatment in the global assessment scale, whereas 46.7% of Group B patients were rated as “excellent”. The differences in Global Assessment ratings between the groups were statistically significant (*p* = 0.000), further supporting the greater efficacy of eperisone-diclofenac in addressing the functional and symptomatic aspects of LBP.

The study by Celik et al. [40] compared the efficacy of medical therapy combining diclofenac and thiocolchicoside (Group I) with zygapophysial joint blockage (Group II) in patients experiencing LBP. A total of 80 participants were randomly divided into two groups and evaluated using the VAS for pain and the ODQ at baseline, the fifth day, and at follow-ups in the first, third, and sixth months. Statistical significance was considered at *p* < 0.05.

At baseline, the mean VAS scores were 7 in Group I and 8 in Group II, reflecting comparable levels of initial pain between groups. On Day 5, VAS scores dropped to 3 in Group I and 2 in Group II, with Group II demonstrating a significantly greater reduction (*p* < 0.001). At one month, VAS scores further reduced to 2 in Group I and 1 in Group II, maintaining statistical significance (*p* < 0.001). By the third month, scores were 4 in Group I and 5 in Group II, with no significant intergroup differences. At six months, VAS scores were 5 in Group I and 2 in Group II. The sustained improvement in Group II compared to Group I was statistically significant (*p* < 0.001).

The ODQ scores at baseline were 21 for Group I and 23 for Group II. On Day 5, ODQ scores improved to 9 in Group I and 5 in Group II, favoring Group II (*p* < 0.001). At one month, scores reduced to 4 for Group I and remained at 5 for Group II, showing no significant intergroup difference. By the third month, scores increased to 7 in Group I and 11 in Group II. At six months, Group I had a score of 11, while Group II improved to 3, demonstrating significantly better long-term outcomes for Group II (*p* < 0.001).

The randomized controlled trial by Iliopoulos et al. [42] investigated the short-term efficacy of a fixed-dose combination (FDC) of diclofenac and thiocolchicoside (test treatment) compared to diclofenac monotherapy (reference treatment) in managing acute moderate-to-severe LBP. A total of 134 patients were enrolled, with 123 completing the per-protocol analysis (62 receiving the test treatment and 61 receiving the reference treatment). Efficacy outcomes focused on changes in pain intensity, assessed via the VAS, and mobility improvement, measured through the FFD test, at baseline and at 1- and 3-h post-administration.

At baseline, the mean VAS scores were 72.03 ± 11.72 mm for the test group and 65.20 ± 12.16 mm for the reference group, with baseline pain intensity significantly higher in the test group (*p* = 0.002). At 1 h, the test group exhibited a mean VAS reduction of 26.66 mm, compared to 16.22 mm in the reference group, with a significant treatment difference of −10.03 mm (95% CI: −15.07 to −4.99; *p* < 0.01). At 3 h, the test group achieved a reduction of 40.47 mm, compared to 20.68 mm in the reference group, yielding a significant treatment difference of −14.65 mm (95% CI: −20.45 to −8.86; *p* < 0.01). A clinically meaningful pain reduction (≥30%) was achieved by 56.5% of patients in the test group and 37.7% in the reference group at 1 h (*p* = 0.037). By 3 h, 91.9% of the test group achieved this threshold compared to 57.4% in the reference group (*p* < 0.01), with the adjusted odds ratio for achieving ≥30% pain reduction at 3 h favoring the test treatment (OR = 8.10, 95% CI: 2.80–23.40).

The mean FFD at baseline was 44.52 ± 17.33 cm in the test group and 41.02 ± 16.12 cm in the reference group, with no significant differences (*p* = 0.249). At 1 h, the test group showed a mean reduction of 12.31 cm, while the reference group reduced by 7.89 cm, with a significant treatment difference of −3.79 cm (95% CI: −7.06 to −0.53; *p* = 0.023). At 3 h, the test group reduced by 19.97 cm, compared to 10.92 cm in the reference group, with a significant treatment difference of −6.91 cm (95% CI: −10.08 to −3.73; *p* < 0.01).

The randomized, double-blind, multicenter study by Landázuri et al. [43] evaluated the efficacy of a fixed-dose combination of thiocolchicoside (4 mg) and potassium diclofenac (50 mg) in patients with acute painful muscle spasms, including cervical, dorsal, and lumbar pain of functional origin. Ninety-seven patients were recruited from three centers in Ecuador and randomized into two groups: an active medication group (n = 50) and a placebo group (n = 47). Efficacy was assessed through visual inspection, palpation, and pain reduction measured on the VAS after five days of treatment.

At baseline, all patients (100%) in both groups exhibited visible muscle spasms. By Day 5, 96% of the active medication group showed no visible signs of muscle spasm, compared to only 2.2% in the placebo group. This difference was statistically significant (*p* < 0.001). Initially, 82% of patients in the active medication group had moderate-to-severe spasms, which decreased to 0% by Day 5. In contrast, the placebo group had 89.1% of patients with moderate-to-severe spasms at baseline, which reduced to 26% by Day 5 (*p* < 0.001).

At baseline, the active group reported an average VAS score of 6.66 cm. By Day 5, the active medication group’s mean VAS score dropped to 0.86 cm. The active medication group achieved a significantly greater reduction in pain (*p* < 0.001), with 90% of patients reporting VAS scores between 0 and 2, compared to only 45.75% in the placebo group.

Paracetamol usage was minimal across groups, with 78% of the active medication group and 72% of the placebo group not requiring additional analgesics. However, statistical analysis excluding patients who used rescue medication did not alter the observed efficacy differences between groups.

The randomized, double-blind, phase III trial conducted by Sproviero et al. [44] evaluated the efficacy and safety of a fixed-dose combination (FDC) of diclofenac sodium (75 mg) and thiocolchicoside (4 mg) administered intramuscularly (IM) compared to separate IM injections of the two components in patients with acute moderate-to-severe LBP. A total of 223 patients were randomized into two groups: the FDC test group (n = 111) and the separate injections reference group (n = 112). The study’s primary efficacy endpoint was the change in pain intensity from baseline, measured using the VAS at rest, 96 ± 2 h after the start of treatment, one hour after the last injection.

Both groups reported comparable baseline VAS scores (Test: 73.55 ± 10.82 mm, Reference: 72.47 ± 11.00 mm). On Day 5, the mean change from baseline was −56.92 ± 18.54 mm in the test group and −55.95 ± 17.29 mm in the reference group. The adjusted least squares mean difference between groups was −0.41 mm (95% CI: −3.98 to 3.16 mm; *p* = 0.82), indicating non-inferiority of the FDC compared to the separate injections. Sensitivity analysis excluding the effect of rescue medication confirmed these results, with a similar mean difference of −0.65 mm (95% CI: −4.24 to 2.94 mm; *p* = 0.72).

Improvements in muscle contracture, assessed by the Schober Index, were comparable in both groups. From baseline to Day 5, the mean change in the distance between the two reference points increased significantly in both groups, demonstrating reduced muscle spasm severity. However, intergroup differences were not statistically significant.

The proportion of patients requiring rescue paracetamol was low and similar between groups (Test: 20.7%, Reference: 19.6%; *p* = 0.84). The cumulative dose of rescue paracetamol used was also not significantly different (Test: 364.9 ± 858.0 mg, Reference: 473.2 ± 1538 mg; *p* = 0.52).

Resolution of pain, defined as a VAS score < 5 mm without subsequent relapse, occurred in 30.4% of the test group and 27.9% of the reference group. The median time to resolution of pain was not calculable due to a low number of resolved cases, and no significant difference was observed between groups (*p* = 0.55).

The prospective study by Akhter and Saddiq [38] evaluated the efficacy of a combination therapy (Diclofenac Sodium 75 mg + Thiocolchicoside 4 mg) compared to Diclofenac Sodium alone in patients with LBP. Conducted in an outpatient orthopedic department, the study spanned August 2016 to August 2017, enrolling 288 patients randomized into two groups (Group A and Group B, n = 144 each). The study’s primary outcomes included changes in pain intensity (measured via VAS) and functional disability (hand-to-floor distance) from baseline (Day 0) to Day 3 and Day 7.

Both groups had comparable mean VAS scores at baseline (Group A: 7.31 ± 0.27, Group B: 7.27 ± 0.26). On Day 3, Group A experienced a significant reduction to 4.10 ± 0.17, while Group B showed a greater reduction to 3.67 ± 0.15 (*p* < 0.0001). On Day 7, VAS scores further decreased in both groups; however, Group B demonstrated a significantly lower score (0.94 ± 0.10) compared to Group A (1.35 ± 0.10, *p* < 0.003).

Both groups had equivalent mean hand-to-floor distances at baseline (Group A: 28.79 ± 0.31 cm, Group B: 28.70 ± 0.28 cm). On Day 3, Group A improved to 18.62 ± 0.26 cm, while Group B exhibited a greater reduction to 16.93 ± 0.12 cm (*p* < 0.0001). On Day 7, the mean distance in Group B reduced significantly to 10.54 ± 0.07 cm, compared to 12.12 ± 0.18 cm in Group A (*p* = 0.0005).

The observational study by Meloncelli et al. [45] compared the efficacy of diclofenac and thiocolchicoside (DIC/THIO) administered intramuscularly (IM) to tramadol-dexketoprofen (TRAM/DKP) taken orally in 82 patients with acute LBP due to lumbar disc herniation. Patients were treated for five days, with pain intensity (PI) measured using a NRS and additional endpoints including the sum of pain intensity difference (SPID), percentage of responders achieving significant pain reduction, and changes in the Douleur Neuropathique (DN4) score for neuropathic pain assessment.

Both treatment groups had comparable baseline NRS scores (TRAM/DKP: 8.8 ± 0.75, DIC/THIO: 8.7 ± 0.75). On Day 3, TRAM/DKP showed significantly greater reductions in PI compared to DIC/THIO (*p* < 0.0001). On Day 7, TRAM/DKP continued to demonstrate superior analgesia, with a larger proportion of patients achieving ≥30% reduction in PI (TRAM/DKP: 95.5%, DIC/THIO: 71.1%, *p* = 0.003).

The mean SPID at Day 7 was significantly higher for TRAM/DKP (770.9 ± 23.5) compared to DIC/THIO (507.1 ± 22.6, *p* < 0.0001). On Day 7, 63.6% of TRAM/DKP patients achieved ≥50% PI reduction compared to 5.3% of those receiving DIC/THIO (*p* < 0.0001).

TRAM/DKP provided a significantly greater reduction in DN4 scores for neuropathic pain from baseline to Day 7 (−62.7 ± 25.6 vs. −39.7 ± 31.2, *p* < 0.0001).

The observational study by Asawari et al. [34] compared the efficacy of diclofenac sodium alone (Group D) versus a combination of diclofenac sodium and thiocolchicoside (Group D + T) in orthopedic patients experiencing musculoskeletal disorders, including LBP. A total of 80 inpatients were divided into two equal groups of 40 each, with pain severity assessed using the VAS at three stages: baseline (admission), during treatment, and at discharge.

Mean VAS scores at admission were similar between the two groups, with Group D reporting 5.35 ± 2.15 and Group D + T reporting 5.27 ± 2.15. At discharge, Group D exhibited a mean VAS score reduction of 4.2 points, while Group D + T achieved a greater reduction of 4.6 points (*p* < 0.05). Pain reduction was more pronounced in Group D + T, with the difference between the two groups being statistically significant (*p* < 0.05).

### 3.5. Synthesis of Efficacy Results

**Figure 4:**
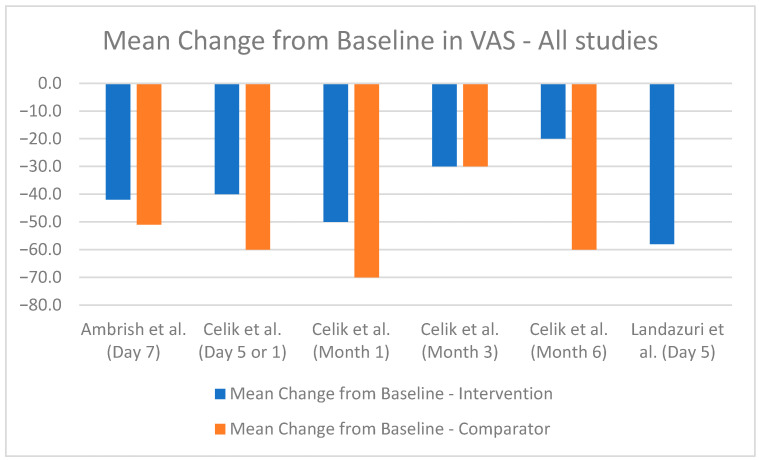
Mean change from baseline in VAS score for oral formulations [40,41,43].

**Figure 5:**
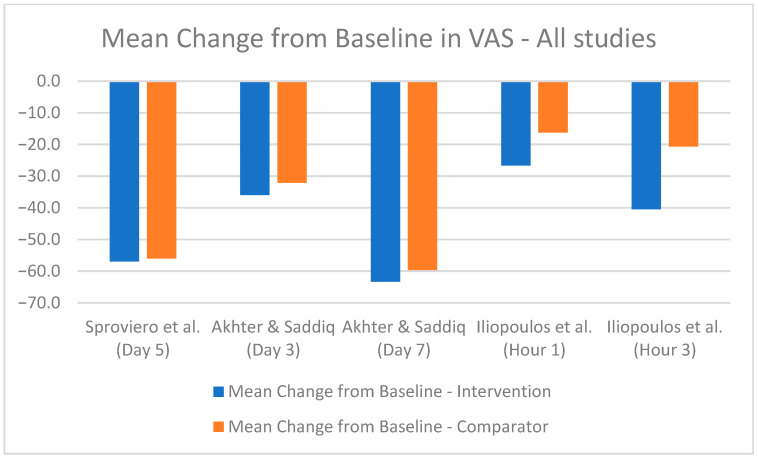
Mean change from baseline in VAS score for intramuscular combinations [38,42,44].

**Figure 6:**
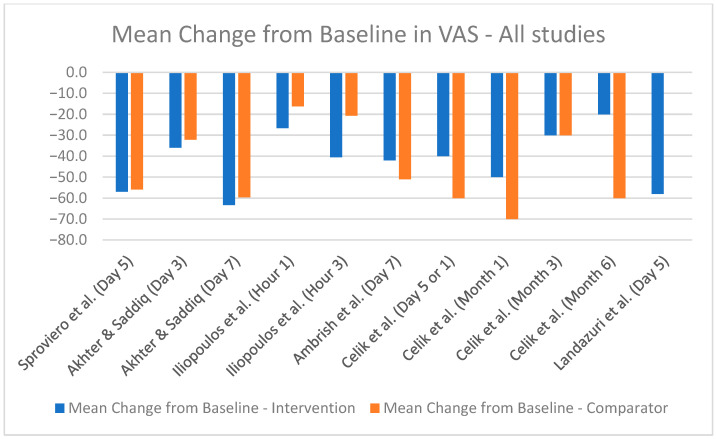
Mean change from baseline in VAS score for all combinations [38,40,41,42,43,44].

The comparative analysis of efficacy outcomes focused primarily on the reduction in pain intensity as measured by the VAS, a common endpoint across most included studies. This uniformity provided a basis for cross-study comparison despite the inherent heterogeneity in study design, interventions, and populations. A bar chart was utilized to visualize the mean change in VAS scores at the most relevant time points for each study, where available (Figure 4, Figure 5 and Figure 6). However, the methodological differences among studies and limited availability of standardized data constrained the potential for statistical synthesis or meta-analysis.

Comparators ranged from monotherapy with diclofenac or thiocolchicoside to alternative therapies such as tramadol-dexketoprofen or zygapophysial joint blockage. This diversity underscores the versatility of the diclofenac and thiocolchicoside combination but also complicates drawing definitive conclusions about its relative superiority.

Most studies demonstrated significant reductions in VAS scores with the combination of diclofenac and thiocolchicoside compared to monotherapy or alternative treatments. Although the VAS score was a unifying measure, the studies varied significantly in baseline pain intensity, treatment durations, and assessment methodologies. This variability limits direct comparability and necessitates cautious interpretation of findings.

Some studies reported VAS scores in centimeters, while others used millimeters. For consistency, Table 4 presents all VAS scores in millimeters. Most studies provided mean baseline VAS scores and mean final VAS scores, allowing for the calculation of the mean difference by subtracting the final score from the baseline score. Only one study directly reported the mean difference. Standard deviations for baseline and final VAS scores were reported in some studies, facilitating precise calculation of the SD of mean differences. However, for most studies, such granular data were unavailable, requiring approximations or exclusion from certain analyses (Table 4). The different study designs and reporting methods further complicate the pooling of data. Therefore, only a simple mean change from baseline could be presented (Figure 4, Figure 5 and Figure 6).

In the context of this review, presenting the change from baseline offers several important contributions, despite the inherent limitations in the available data. By calculating and reporting mean changes in VAS scores from baseline, the review establishes a uniform measure of treatment effectiveness across studies. The use of change from baseline allows for a simplified interpretation of efficacy. It provides a direct and straightforward assessment of the intervention’s impact on pain reduction, which is easily understood without requiring complex statistical adjustments. This approach also broadens the range of studies that can be included, ensuring that valuable data is not excluded due to incomplete reporting.

However, the use of changes from baseline is not without its limitations. This approach does not provide insights into the relative efficacy of interventions compared to placebo or active comparators, as it does not account for differences between groups. Additionally, baseline imbalances between groups may bias the interpretation of changes, especially in the absence of statistical adjustments for confounders. The lack of reported standard deviations or confidence intervals also limits the ability to assess the statistical significance or reliability of the findings.

### 3.6. Safety Outcomes

Only four out of nine studies included in this systematic review investigated both the efficacy and the safety and tolerability of the combination of Diclofenac and Thiocolchicoside (Table 5). Their findings are presented below.

Meloncelli et al. [45] found that both treatment groups demonstrated good tolerability. Treatment-Emergent Adverse Events (TEAEs) were reported in 7.9% in the DIC/THIO group. Importantly, no serious AEs occurred, and no patients discontinued treatment due to TEAEs. In the DIC/THIO group, injection site pain was the only treatment-related TEAE observed. Additionally, no epileptic seizure events were reported, as patients with known hypersensitivity to thiocolchicoside were excluded.

Landázuri et al. [43] demonstrated that the combination of thiocolchicoside (4 mg) and potassium diclofenac (50 mg) administered twice daily for five days had a favorable safety profile. AEs were mild and reported in 8% of the active medication group, compared to 17.4% in the placebo group. In the active group, adverse reactions included epigastralgia, gastritis, and dizziness, while the placebo group reported headache, somnolence, arm tremors, hypotension, and GI issues. Importantly, no patients in the active group discontinued treatment due to AEs, whereas 10.9% of the placebo group withdrew from the study due to either insufficient efficacy or adverse reactions.

Additionally, the active medication did not significantly affect psychomotor performance or alertness, as evidenced by psychometric tests and somnolence assessments. Scores for somnolence remained low throughout the study, with no statistical differences observed between the active and placebo groups. The medication also exhibited excellent GI tolerability, with no cases of diarrhea, often linked to lactose-containing formulations, since the study tablet did not include lactose. Overall, the combination therapy was safe, well-tolerated, and suitable for managing acute painful muscle spasms.

In Sproviero et al. [44], TEAEs were reported in 5.4% of patients in the fixed-dose combination group and 6.3% in the separate injection group, with treatment-related TEAEs observed in 2.7% and 0.9% of patients, respectively. The most common TEAEs included mild abnormalities in laboratory parameters and nausea. Importantly, no serious AEs occurred in either group, and no patients discontinued treatment due to AEs. The findings suggest the fixed-dose combination is as safe as administering the two drugs separately, with comparably low rates of adverse effects.

No AEs were reported by Iliopoulos et al. [42] in the group receiving the combination treatment. In contrast, the reference group, which received diclofenac monotherapy, experienced two cases of mild dizziness. These instances occurred shortly after administration and resolved spontaneously without intervention. Importantly, no patients in either group withdrew from the study due to AEs, highlighting the excellent tolerability of the fixed-dose combination. These findings reinforce the safety and suitability of this therapeutic option for managing acute LBP.

### 3.7. Reporting Bias and Certainty of Evidence

Following the GRADE approach, the overall certainty of evidence for the evaluable outcome of pain reduction as assessed using the VAS for the combination of Diclofenac and Thiocolchicoside was judged to be low. This was primarily due to the high risk of bias exhibited by most of the studies included. As for the inconsistency, a minimally important difference (MID) threshold was used to assess whether a clinically important effect was present. Different MID thresholds for VAS have been proposed. For this review, an MID of 10 mm was used in line with validation studies. In studies that compared the change difference between the intervention and the comparator, the results for the mean change in VAS scores were systematically lower than the MID, with the exception of the study conducted by Iliopoulos et al. [42]. However, changes from baseline were always much higher than the MID. Overall, a more conservative approach was followed, and the quality of evidence was rated down for inconsistency, mainly due to the heterogeneity in study designs. Indirectness was not an issue in any of the studies included, as they all investigated the combination of interest in samples deriving from the general population or interest (i.e., patients with LBP). In addition, a high level of imprecision was detected, as most studies had small sample sizes and failed to report the standard deviation values, whereas studies that did present limits reported high confidence intervals. Finally, given that the number of studies included in this review was small, publication bias could not be assessed using funnel plots, nor formally tested through Egger’s test. However, a potential publication bias was found due to the presence of registered and completed RCTs, whose results were not published (e.g., EudraCT Numbers 2022-000724-37 and 2015-002476-24) [46,49].

The certainty of the evidence for pain reduction using the VAS was downgraded due to multiple concerns. First, some included studies, particularly observational ones, exhibited a high risk of bias due to variability in study designs, randomization, and blinding. This raises concerns about the reliability of their findings. Second, there was notable inconsistency in the results across studies, with varying magnitudes of effect that were not entirely explained by differences in populations or interventions. Third, imprecision was evident as several studies had small sample sizes and lacked consistently reported confidence intervals, limiting the robustness of effect estimates. Finally, potential publication bias was identified as a concern due to the reliance on published studies, which may selectively report favorable results. Collectively, these factors led to downgrading the certainty of evidence to low.

Downgrades:

- Risk of Bias: −1
- Inconsistency: −1
- Indirectness: 0
- Imprecision: −1
- Publication Bias: −1

## 4. Discussion

The findings of the present systematic review provide insights into the efficacy and safety of the combination therapy of diclofenac and thiocolchicoside for managing LBP. While the data suggest significant pain relief and functional improvement, methodological limitations and heterogeneity across studies warrant cautious interpretation.

Several studies have explored the combination of NSAIDs and muscle relaxants for managing LBP, aiming to leverage the anti-inflammatory effects of NSAIDs and the spasm-relieving properties of muscle relaxants [11,28]. However, the findings across these studies are heterogeneous, with varying efficacy outcomes and safety profiles. This heterogeneity can be attributed to differences in study designs, populations, and the specific substances examined within each drug class [11,28,50,51]. For instance, some studies focus on cyclobenzaprine, a centrally acting muscle relaxant [52,53], while others evaluate thiocolchicoside or tizanidine [54]. Similarly, the choice of NSAID, ranging from ibuprofen [52] to diclofenac, introduces variability in both efficacy and AE profiles. The mixed results are further compounded by inconsistencies in outcome measures, such as pain reduction, functional improvement, and AEs. Some studies have reported synergistic effects, leading to enhanced pain relief and faster recovery, whereas others found only marginal benefits over monotherapy, often accompanied by an increased risk of AEs, including GI discomfort and CNS effects. Furthermore, many trials have methodological limitations, such as small sample sizes, short follow-up durations, or inadequate control of confounding factors, which constrain the generalizability of their findings [11,28,50,51].

Nevertheless, the combination of NSAIDs and muscle relaxants has shown greater efficacy than either drug alone. One study found that patients receiving NSAIDs combined with muscle relaxants experienced a more significant reduction in disability index compared to those on NSAIDs alone [55]. Similarly, a systematic review highlighted that several muscle relaxants are effective for musculoskeletal pain [51]. The combination of diclofenac’s anti-inflammatory properties and thiocolchicoside’s muscle relaxant effects appears to offer symptom relief. However, direct comparisons with alternative treatments revealed only marginal superiority or equivalence, raising questions about its clinical advantage over other available options.

Beyond traditional muscle relaxants, the combination of NSAIDs with B vitamins has also shown promising results. A meta-analysis revealed that diclofenac combined with thiamine, pyridoxine, and cyanocobalamin significantly reduced treatment duration compared to diclofenac alone in patients with low back pain, suggesting a synergistic effect that enhances analgesic efficacy [56]. It has been demonstrated that this combination improves mobility and pain scores more effectively than diclofenac monotherapy [57]. Alternative combinations involving tramadol, an atypical opioid, also offer benefits for LBP management. One study found that tramadol/acetaminophen combination tablets provided significant pain relief, improved physical functioning, and enhanced quality of life compared to placebo [58]. More recently, another study reported that etoricoxib combined with tramadol delivered faster pain relief, better adherence due to once-daily dosing, and reduced opioid dependency compared to paracetamol/tramadol [59]. These findings underscore the potential of multimodal analgesia, where combining drugs with different mechanisms can achieve better outcomes with lower doses, thus minimizing side effects.

Beyond pharmacological strategies, multidisciplinary interventions are increasingly recognized as effective in CLBP management. A recent Bayesian network meta-analysis by Baroncini et al. [60] identified Adapted Physical Exercise (APE), Physical Agent Modalities, and a Multidisciplinary Approach as among the most effective strategies for pain and disability reduction in CLBP. Given that Diclofenac + Thiocolchicoside reduces inflammation and muscle spasticity, it is plausible that its use alongside physiotherapy-based approaches could enhance functional recovery by improving tolerance to rehabilitative exercises. However, muscle relaxants may also temporarily affect neuromuscular control, potentially influencing movement-based therapies.

Additionally, another recent systematic review by Migliorini et al. [61] found that educational interventions did not significantly improve pain or disability outcomes when added to physiotherapy in CLBP patients. This underscores the need for further research into whether a combined approach integrating pharmacotherapy with structured physiotherapy regimens offers superior outcomes compared to either strategy alone.

Safety data, though limited, indicate a generally favorable profile with mild adverse events, such as gastrointestinal discomfort and drowsiness, in our study. However, the absence of long-term safety assessments limits conclusions regarding prolonged use. It is important to note that only four of the studies included in the present review explicitly assessed both efficacy and safety outcomes, while the remaining studies primarily focused on pain relief and functional improvement. This limited number of safety evaluations restricts the robustness of our conclusions regarding the adverse event profile of the Diclofenac + Thiocolchicoside combination. This underscores the importance of careful patient selection and monitoring, particularly in populations at risk for gastrointestinal, cardiovascular, or neurological complications. In this context, most combinations maintain a favorable profile. For instance, a study observed that tizanidine combined with ibuprofen not only provided faster pain relief but also resulted in fewer gastrointestinal side effects compared to ibuprofen alone. However, the combination was associated with sedation, which, while advantageous for severe acute pain, may limit its use in certain populations [62]. Adverse effects such as dry mouth, dizziness, and muscle weakness were noted with specific muscle relaxants, particularly tizanidine and baclofen [51]. Nonetheless, the incidence of serious side effects, such as hepatotoxicity, was rare and primarily associated with dantrolene and chlorzoxazone [51].

The systematic approach employed in this review strengthens its reliability, with a clear eligibility framework, dual-reviewer data extraction, and structured bias assessment using RoB 2 and the NOS. The inclusion of both RCTs [38,39,40,41,42,43,44] and observational studies [34,45] offers a comprehensive view of the evidence, balancing controlled experimental data with real-world clinical outcomes. The broad literature search, without language restrictions, minimizes the risk of missing relevant studies.

Nevertheless, several limitations must be acknowledged. Our search strategy was limited to two primary databases (PubMed and Scopus). These were chosen due to their comprehensive coverage of biomedical and clinical literature. While additional databases such as CINAHL, PEDro, and Web of Science could have contributed to a broader dataset, we prioritized databases most relevant to pharmacological and clinical studies. Additionally, we did not conduct manual backward citation searching (reference list screening) of included studies, which might have identified additional relevant articles. However, our structured search approach ensured a systematic identification of the most relevant studies. Furthermore, while we imposed no language restrictions, our search terms were in English, which may have inherently favored English-language studies and introduced a potential language bias. Although major databases index studies in multiple languages, some non-English studies might not have been retrieved. Finally, ResearchGate and Academia.edu were searched to locate published journal articles indexed on these platforms. Only formally published studies meeting our inclusion criteria were considered, and we acknowledge that the inability to quantify search results from these sources introduces some degree of uncertainty in study selection reporting.

The high heterogeneity across studies, including differences in patient demographics, baseline pain severity, and comparator treatments, complicates direct comparisons and precludes meta-analysis. Variability in assessment tools, such as VAS, ODI, and other functional measures, further challenges uniform interpretation. Some studies also exhibited a high risk of bias due to unclear randomization procedures [38,40,41,43], inadequate blinding, or missing data handling [39,40,43], potentially impacting result reliability. Additionally, small sample sizes in many trials limit statistical power, while short follow-up periods [42] restrict insights into long-term efficacy and safety. Potential publication bias remains a concern, as some completed RCTs on this combination therapy have not been published. The absence of negative or inconclusive results in the available literature raises the possibility of selective reporting. Furthermore, while the review provides valuable insights into acute pain management, evidence supporting the combination therapy for CLBP is lacking.

Despite these limitations, our systematic and structured methodology, adherence to PRISMA guidelines, and risk-of-bias assessments strengthen the reliability of our findings. Future research should consider expanding database coverage, incorporating reference list screening, and formally assessing the impact of language restrictions to further enhance the robustness of evidence synthesis.

## 5. Conclusions

The systematic review provides a comprehensive evaluation of the efficacy and safety of the combination therapy of diclofenac and thiocolchicoside in the management of LBP and related musculoskeletal conditions. The findings demonstrate that this combination offers significant pain relief and functional improvement, particularly in acute cases, owing to the synergistic effects of its anti-inflammatory and muscle-relaxant properties. However, the overall quality of evidence is limited by methodological heterogeneity, small sample sizes, and short study durations.

While the combination therapy shows promise as an effective option for acute LBP, its role in chronic pain management remains unsubstantiated due to insufficient long-term data. The therapy’s safety profile necessitates caution, particularly for patients with GI, cardiovascular, or neurological risks. This highlights the importance of careful patient selection, adherence to short-term use, and close monitoring during treatment.

In clinical practice, this combination therapy may be considered for acute cases of LBP, particularly where muscle spasm is a dominant feature. However, clinicians must exercise judgment, balancing its potential benefits against known risks and tailoring treatment to individual patient needs.

In conclusion, the combination of diclofenac and thiocolchicoside represents a viable option for acute pain management within specific contexts. Nevertheless, more rigorous and standardized research is essential to confirm its efficacy and safety, guide clinical decision-making, and inform evidence-based recommendations.

## Figures and Tables

**Table 1:** Summary of the main eligibility criteria.

Element	Eligibility Criteria
Population (P)	Adults (≥18 years) diagnosed with LBP or other musculoskeletal conditions for which Diclofenac + Thiocolchicoside is indicated. No restrictions on sex, ethnicity, or comorbidities.
Intervention (I)	Combined use of Diclofenac and Thiocolchicoside, administered via any route (oral, intramuscular, or other formulations) and at any dosage.
Comparison (C)	Eligible comparators included: (1) Diclofenac monotherapy, (2) Thiocolchicoside monotherapy, (3) Other treatments for LBP or musculoskeletal pain, (4) Placebo, (5) No treatment.
Outcome (O)	Primary Outcomes: Pain relief, functional improvement, and patient-reported quality of life. Secondary Outcomes: Incidence and severity of AEs associated with the combination therapy.

**Table 2:** Risk of bias in observational studies.

Study	Selection	Comparability	Outcome	Total
Meloncelli et al. [45]	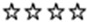	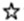	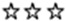	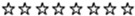
Asawari et al. ^1^ [34]	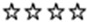	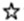	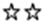	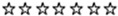

^1^ Assessed without considering Beall’s List.

**Table 3:** Summary of efficacy outcome measures in RCTs.

Study	Celik et al. [40]	Landázuri et al. [43]	Ambrish et al. [41]	Sproviero et al. [44]	Altan et al. [39]	Iliopoulos et al. [42]	Akhter & Siddiq [38]
ODQ/ODI	√	-	-	-	√	-	-
VAS	√	√	√	√	-	√	√
VNS	-	-	-	-	√	-	-
FFD	-	-	√	-	-	√	√
Schober test	-	-	-	√	√	-	-
Visual Inspection	-	√	-	-	-	-	-
Palpation	-	√	-	-	-	-	-
Rescue medication	-	√	-	-	-	-	-
Treatment satisfaction	-	√	-	-	-	-	-
Lasegue’s sign	-	-	√	-	-	-	-
Global Assessment of response to therapy	-	-	√	-	-	-	-
Safety	-	√	-	√	-	√	-

ODQ/ODI: Oswestry Disability Questionnaire/Oswestry Disability Index, VAS: Visual Analogue Scale, VNS: Visual Numeric Scale.

**Table 4:** VAS Scores reported by dosage form.

Study	Diclofenac + Thiocolchicoside Intervention		Comparator		Timepoint
	Mean ± SD	Mean Change ± SD (From Baseline)	Mean ± SD	Mean Change ± SD (From Baseline)	
Oral Intervention					
Ambrish et al. [41]	63.7 ± 16.3	NA	64.3 ± 17.9	NA	Baseline
	21.7 ± 13.1		13.3 ± 16.3		Day 7
Celik et al. [40]	70		80		Baseline
	30		20		Day 5 or Day 1
	20		10		Month 1
	40		50		Month 3
	50		20		Month 6
Landázuri et al. [43]	66.6		NA		Baseline
	8.6				Day 5
Intramuscular Intervention					
Sproviero et al. [44]	NA	−56.92 ± 18.54	NA	−55.95 ± 17.29	Day 5
Akhter & Saddiq [38]	72.7	NA	73.1	NA	Baseline
	36.7		41		Day 3
	9.4		13.5		Day 7
Iliopoulos et al. [42]	72.03 ± 11.72	NA	65.20 ± 12.16	NA	Baseline
	45.37 ± 16.28		48.98 ± 18.76		Hour 1
	31.56 ± 15.08		44.52 ± 17.33		Hour 3

**Table 5:** PICO table of included studies.

Author (Publication Year)	Population (P)	Intervention (I)	Comparator (C)	Outcome (O)
Celik et al. (2011) [40]	Adults 20–60 years old with low back pain (max 4-month history of pain)	Oral Diclodenac sodium 100 mg/day for 5 days + Oral Thiocolchicoside 8 mg/day for 5 days + bed rest for 4 days	Bilateral L4/5 and L5/ S1 zygapophysial joints blockage percutaneously with 22 G spinal needle by prilocaine 10 mg bupivacaine 5 mg and methylprednislone acetate under PA and lateral fluoroscopy	Zygapophysial joint blockage (Group II) outperformed medical therapy (Group I) in reducing VAS and ODQ scores at Day 5 and 6 months (*p* < 0.001). No significant differences at 1 month for ODQ. Group II reported superior and sustained pain relief and functional improvement, with practical advantages (no bed rest needed).
Landázuri et al. (2015) [43]	Adults 18–60 years old with low back pain, dorsalgias or cervicalgias	Oral fixed dose combination tablet of Diclofenac + Thiocolchicoside (50 + 4) mg/tab twice a day for 5 days	Placebo	Active group (thiocolchicoside + potassium diclofenac) showed >85% pain reduction (VAS) and near-complete resolution of muscle spasms (*p* < 0.001). Mild adverse events in 8% of active group compared to 17.4% in placebo group. No treatment discontinuations in active group. Significant reduction in muscle spasms and pain, with low need for rescue medication in the active group.
Ambrish et al. (2017) [41]	Adults 20–60 years old with low back pain of acute onset	Oral Diclofenac 50 mg + oral Thiocolchicoside 4 mg twice a day for 7 days	Oral Esperidone sustained release 150 mg + oral diclofenac 100 mg once a day for 7 days	Eperisone-diclofenac group (Group B) showed greater improvements in FFD, Lasegue’s Sign, VAS, and Global Assessment Scale compared to thiocolchicoside-diclofenac group (Group A) (*p* < 0.05). Higher satisfaction reported in Group B (greater improvement in therapeutic response).
Altan et al. (2019) [39]	Adults 20–50 years old with low back pain (max 12 weeks history of pain)	Diclofenac + Thiocolchicoside gel + ultrasound	Conventional ultrasound	Significant reduction in VNS scores at rest and during movement (*p* < 0.05). Greater ODI improvement in Group 1 (diclofenac-thiocolchicoside gel via PP). No significant differences in lumbar flexibility improvement between groups. Greater functional improvement and pain relief reported in Group 1, supporting combination therapy for ALBP.
Sproviero et al. (2018) [44]	Adults 18–65 years old with acute low back pain (max 7 days onset) of moderate to severe intensity (≥5 cm VAS)	IM fixed dose combination tablet of Diclofenac + Thiocolchicoside (75 + 4) mg/4 mL once a day for 5 days	IM Diclofenac (Voltaren) 75 mg + IM Thiocolchicoside (Muscoril) 4 mg for 5 days	FDC group and separate injection group showed similar pain reduction and muscle contracture relief. Non-inferiority of FDC established (*p* = 0.82). Low incidence of TEAEs in both groups (5.4% in FDC group, 6.3% in reference group). No serious adverse events reported. Comparable patient satisfaction and compliance in both groups, with FDC offering convenience of fewer injections.
Iliopoulos et al. (2023) [42]	Adults with acute low back pain (max 7 days onset) of moderate to severe intensity (≥40 mm VAS)	IM fixed dose combination tablet of Diclofenac + Thiocolchicoside (75 + 4) mg/4 mL as one signle dose	IM Diclofenac (Voltaren) 75 mg as one single dose	Fixed-dose combination (FDC) group had greater and faster reduction in VAS scores and mobility improvement (*p* < 0.01) compared to diclofenac monotherapy. 91.9% achieved clinically meaningful pain reduction at 3 h in FDC group. No adverse events in FDC group; mild dizziness reported in diclofenac monotherapy group. FDC group reported rapid pain relief and improved mobility within 3 h.
Akhter and Siddiq (2017) [38]	Adults with acute and subacute low back pain treated at an orthopedic outpatient department	IM Diclofenac Sodium 75 mg twice daily + IM Thiocolchicoside 4 mg twice daily for 7 days	IM Diclofenac Sodium 75 mg twice daily for 7 days	Combination therapy group (Group B) showed superior pain relief (VAS) and functional improvement (hand-to-floor distance) compared to monotherapy (Group A) by Day 7 (*p* < 0.05). Combination therapy yielded better pain relief and functional mobility improvement, with good tolerability reported.
Asawari et al. (2013) [34]	Adult patients with musculoskeletal pain admitted in an orthopedic ward	Diclofenac 75 mg + Thiocolchicoside 4 mg (Group D + T) twice a day	Diclofenac 75 mg (Group D) twice a day	Combination therapy (Group D + T) led to significantly greater pain reduction (VAS) compared to monotherapy (Group D) (*p* < 0.05). Combination therapy demonstrated better therapeutic outcomes, supporting its use for musculoskeletal pain.
Meloncelli et al. (2020) [45]	Adult patients with lumbar disk herniation and acute radicular pain	Oral Tramadol 75 mg + Dexketoprofen 25 mg (TRAM/DKP)	IM Diclofenac 75 mg + Thiocolchicoside 4 mg (DIC/THIO)	Tramadol-dexketoprofen (TRAM/DKP) group outperformed diclofenac-thiocolchicoside (DIC/THIO) in pain reduction, responder rates, and neuropathic pain (*p* < 0.0001). TEAEs in 18.2% of TRAM/DKP group and 7.9% of DIC/THIO group. No serious adverse events; TEAEs resolved by Day 7. Higher pain relief and improved neuropathic pain in TRAM/DKP group, highlighting greater efficacy than DIC/THIO.

ODQ/ODI: Oswestry Disability Questionnaire/Oswestry Disability Index, VAS: Visual Analogue Scale, VNS: Visual Numeric Scale, FFD: Finger-to-Floor Distance, FDC: Fixed-Dose Combination, TEAE: Treatment-Emergent Adverse Event, TRAM/DKP: Tramadol-Dexketoprofen, DIC/THIO: Diclofenac-Thiocolchicoside.

## Data Availability

Not applicable.

## Supplementary Materials

The following supporting information can be downloaded at: https://www.mdpi.com/article/10.3390/healthcare13060677/s1, Risk of bias assessment for RCTs and observational studies [34,35,36,38,39,40,41,42,43,44,45,63].

## Abbreviations

The following abbreviations are used in this manuscript:

VAS	Visual Analogue Scale
WHO	World Health Organization
LBP	Low Back Pain
NSAID	Non-Steroidal Anti-Inflammatory Drug
CLBP	Chronic Low Back Pain
COX	Cyclooxygenase
GABAA	Gamma-Aminobutyric Acid Type A Receptor
API	Active Pharmaceutical Ingredient
EU	European Union
RCT	Randomized Controlled Trial
RoB2	Cochrane Risk of Bias 2 Tool
NOS	Newcastle-Ottawa Scale
GRADE	Grading of Recommendations, Assessment, Development, and Evaluations
ODI	Oswestry Disability Index
ODQ	Oswestry Disability Questionnaire
IM	Intramuscular
AE	Adverse Event
ITT	Intention-To-Treat
VNS	Visual Numeric Scale
MRI	Magnetic Resonance Imaging
NRS	Numerical Rating Scale
FFD	Finger-to-Floor Distance
FDC	Fixed-Dose Combination
SPID	Sum of Pain Intensity Difference
DN4	Douleur Neuropathique score
SD	Standard Deviation
CI	Confidence Interval
TEAE	Treatment-Emergent Adverse Event
MID	Minimally Important Difference
GI	Gastrointestinal
CNS	Central Nervous System
