# Supplementary material for: Efficacy and Safety of the Combination of Diclofenac and Thiocolchicoside in the Treatment of Low Back Pain and Other Conditions: Systematic Review of the Literature
Source: Healthcare (Basel). 2025 Mar 20;13(6):677. doi: 10.3390/healthcare13060677 (PMC11942599; doi:10.3390/healthcare13060677)
Supplement: Supplementary file 1 [file healthcare-13-00677-s001.zip › healthcare-3519091-supplementary.pdf]

healthcare

MDPI

Supplementary Material

Efficacy and Safety of the Combination of Diclofenac and Thiocolchicoside in the Treatment of Low Back Pain and Other Conditions: Systematic Review of the Literature

Ioannis Oikonomou [1,*] and Karolina Akinosoglou [1,2]

[1] Department of Medicine, University of Patras, 26504 Rio, Greece; akin@upatras.gr
[2] Department of Internal Medicine and Infectious Diseases, University General Hospital of Patras, 26504 Rion, Greece
* Correspondence: jc.economou@gmail.com

Supplementary Material: Risk of bias assessment for RCTs and observational studies

The included RCTs were assessed using the RoB 2 tool[1], evaluating five domains: (1) randomization, (2) deviations from intended interventions, (3) missing outcome data, (4) outcome measurement, and (5) selection of reported results.

Domain 1: Randomization Process

Studies by Sproviero *et al*.[2], Iliopoulos *et al*.[3], and Altan *et al*.[4] demonstrated low risk of bias, utilizing adequate randomization methods and allocation concealment. However, three studies (Landázuri *et al*.[5], Celik *et al*.[6], Akhter & Siddiq[7]) had some concerns due to unclear or insufficient reporting of randomization procedures. One study (Ambrish *et al*.[8]) had a high risk of bias due to lack of allocation concealment and baseline imbalances that were not adequately adjusted for in analysis.

Domain 2: Deviations from Intended Interventions

Double blinding in studies such as Sproviero *et al*.[2] and Landázuri *et al*.[5] minimized performance bias. Conversely, open-label studies (Ambrish *et al*.[8], Celik *et al*.[6]) introduced a moderate to high risk of bias due to potential deviations in treatment adherence. One single-blind study (Iliopoulos *et al*.[3]) employed a per-protocol analysis rather than intention-to-treat, raising concerns about potential bias.

Domain 3: Missing Outcome Data

Sproviero *et al*.[2], Landázuri *et al*.[5], and Ambrish *et al*.[8] had minimal missing data, ensuring a low risk of bias. In contrast, studies such as Altan *et al*.[4] and Celik *et al*.[6] had substantial dropout rates without sufficient methods for handling missing data, leading to a high risk of bias. A high risk was also identified for Akhter & Siddiq[7] due to lack of transparency.

Domain 4: Outcome Measurement

Low risk of bias was observed in Altan *et al*.[4], Landázuri *et al*.[5], and Sproviero *et al*.[2], where validated assessment tools and blinding of assessors were employed. However, in Iliopoulos *et al*.[3], Celik *et al*.[6] and Ambrish *et al*.[8], outcome assessors were aware of treatment

assignments, increasing the potential for measurement bias. A high risk was also identified for Akhter & Siddiq[7] due to lack of transparency.

Domain 5: Selection of Reported Results

Studies such as Iliopoulos *et al*.[3] and Sproviero *et al*.[2] demonstrated adherence to pre-specified analysis plans. However, others, including Celik *et al*.[6] and Ambrish *et al*.[8], lacked transparency in reporting, raising concerns about selective outcome reporting and potential data-driven analysis.

Overall Risk of Bias in RCTs

Sproviero *et al*.[2] emerged as the most methodologically robust study with consistently low risk of bias. Iliopoulos *et al*.[3] and Landázuri *et al*.[5] had some concerns but maintained methodological rigor in key areas. Other studies exhibited significant shortcomings, particularly in allocation concealment, blinding, and handling of missing data, which undermined the reliability of their findings.

Observational studies were assessed using the NOS[9], focusing on selection, comparability, and outcome assessment.

The retrospective cohort study by Meloncelli *et al*.[10] demonstrated a low risk of bias, with well-defined exposure groups, validated pain assessment tools, and minimal loss to follow-up. Some concerns existed regarding the representativeness of the exposed cohort, which included only patients with MRI-confirmed lumbar disc herniation.

The prospective cohort study by Asawari *et al*.[11] with moderate risk of bias due to its quasi-randomization approach, lack of baseline stratification, and insufficient statistical adjustments for confounders. Outcome reporting lacked clarity, and comparisons between groups were not explicitly tested for statistical significance. Additionally, concerns arose due to its publication in a potentially predatory journal[12].

Overall Risk of Bias in Observational Studies

While Meloncelli *et al*. [10] provided relatively robust evidence with minor limitations, Asawari *et al*. [11] exhibited several methodological weaknesses, particularly in group comparability and statistical transparency. These findings emphasize the need for rigorous design and comprehensive reporting in observational research to enhance the reliability of evidence.

References

1. Sterne, A.; Savović, J.; Page, M.; Elbers, R.; Blencowe, N.; Boutron, I.; Cates, C.; Cheng, H.-Y.; Corbett, M.; Eldridge, S.; et al. RoB 2: A Revised Tool for Assessing Risk of Bias in Randomised Trials 2019.
2. Sproviero, E.; Albamonte, E.; Costantino, C.; Giossi, A.; Mancuso, M.; Rigamonti, A.; Tornari, P.; Caggiano, G. Efficacy and Safety of a Fixed Combination of Intramuscular Diclofenac 75 Mg + Thiocolchicoside 4 Mg in the Treatment of Acute Low Back Pain: A Phase III, Randomized, Double Blind, Controlled Trial. *Eur J Phys Rehabil Med* **2018**, *54*, doi:10.23736/S1973-9087.17.04923-1.
3. Iliopoulos, K.; Koufaki, P.; Tsilikas, S.; Avramidis, K.; Tsagkalis, A.; Mavragani, C.; Zintzaras, E. A Randomized Controlled Trial Evaluating the Short-Term Efficacy of a Single-Administration Intramuscular Injection with the Fixed Combination of Thiocolchicoside-Diclofenac versus Diclofenac Monotherapy in Patients with Acute Moderate-to-Severe Low Back Pain. *BMC Musculoskelet Disord* **2023**, *24*, 476, doi:10.1186/s12891-023-06599-0.
4. Altan, L.; Kasapoğlu Aksoy, M.; Kösegil Öztürk, E. Efficacy of Diclofenac & Thiocolchioside Gel Phonophoresis Comparison with Ultrasound Therapy on Acute Low

Back Pain; a Prospective, Double-Blind, Randomized Clinical Study. *Ultrasonics* **2019**, *91*, 201–205, doi:10.1016/j.ultras.2018.08.008.

5. Landázuri, D.I.G.; Vargas, D.O.P.; Jaime, D.; Obando, M.; Ponce, D.C.B.; Gonzalez, D.E.M.; Burgos, D.G.G. Estudio controlado, doble ciego, randomizado, multicéntrico, para evaluar la eficacia de la combinación fija tiocolchicósido más diclofenaco potásico en contracturas. **2015**.
6. Celik, B.; Er, U.; Simsek, S.; Tibet, A.; Murad, B. Effectiveness of Lumbar Zygapophysial Joint Blockage for Low Back Pain. *Turkish Neurosurgery* **2011**, doi:10.5137/1019-5149.JTN.4057-10.1.
7. Akhter, N.; Zahid Siddiq, M. Comparative Efficacy of Diclofenac Sodium Alone and in Combination with Thiocolchicoside in Patients with Low Back Pain | Cochrane Library Available online: https://www.cochranelibrary.com/central/doi/10.1002/central/CN-01441517/full (accessed on 5 January 2025).
8. Ambrish, S.; Nagesh, R.; Dharmaraj, B.; Nagendra, G. A Comparative Study of Efficacy of Thiocolchicoside with Diclofenac vs Eperisone with Diclofenac in Patients with Back Pain. *Int J Surg Ortho* **2017**, *3*, 1–5, doi:10.17511/ijoso.2017.i01.01.
9. Wells, G.; Shea, B.; O'Connell, D.; Peterson, J.; Welch, V.; Tugwell, P. The Newcastle-Ottawa Scale (NOS) for Assessing the Quality of Nonrandomised Studies in Meta-Analyses. **2000**.
10. Meloncelli, S.; Divizia, M.; Germani, G. Efficacy and Tolerability of Orally Administered Tramadol/Dexketoprofen Fixed-Dose Combination Compared to Diclofenac/Thiocolchicoside in Acute Low Back Pain: Experience from an Italian, Single-Centre, Observational Study. *Current Medical Research and Opinion* **2020**, *36*, 1687–1693, doi:10.1080/03007995.2020.1814228.
11. Asawari, R.; Goutham, R.; Sanjay, P.; Nitin, D. COMPARATIVE EFFICACY OF COMBINED USE OF DICLOFENAC WITH THIOCOLCHICOSIDE AND DICLOFENAC ALONE IN ORTHOPEDIC PATIENTS. **2013**.
12. Standalone Journals – Beall's List 2025.
